# Supplementary material for: Horizontal Gene Transfers Underpin Ribose Heterotrophy and Central Carbon Metabolism Remodeling in Gloeobacteraceae
Source: Genome Biol Evol. 2026 May 23;18(6):evag127. doi: 10.1093/gbe/evag127 (PMC13237429; doi:10.1093/gbe/evag127)
Supplement: evag127_Supplementary_Data [file evag127_supplementary_data.zip › Figures_Suppl.pdf]

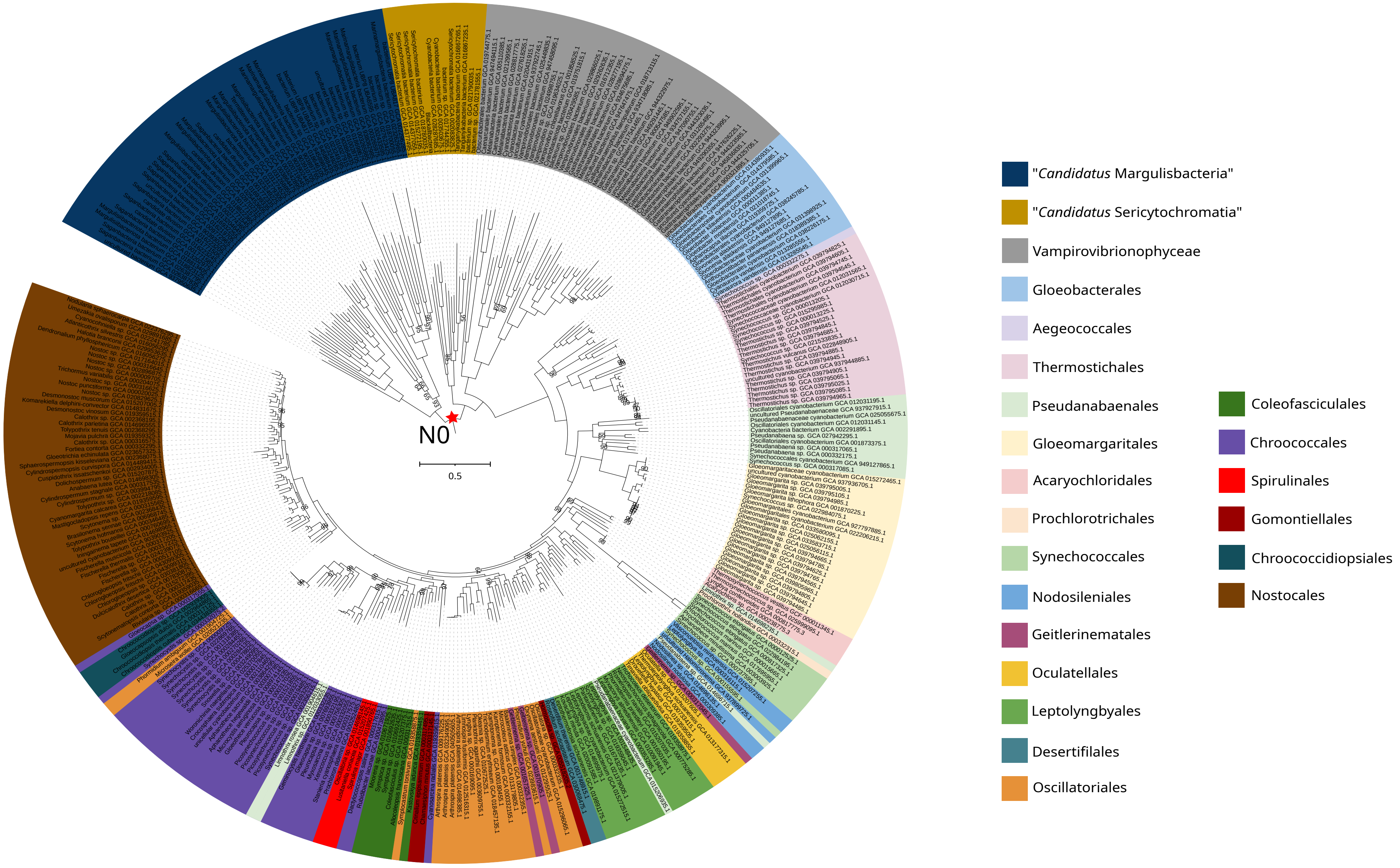
N0
0.5
"Candidatus Margulisbacteria"
"Candidatus Sericytochromatia"
Vampirovibrionophyceae
Gloeobacterales
Aegeococcales
Thermostichales
Pseudanabaenales
Gloeomargaritales
Acaryochloridales
Prochlorotrichales
Synechococcales
Nodosileniales
Geitlerinematales
Oculatellales
Leptolyngbyales
Desertifilales
Oscillatoriales
Coleofasciculales
Chroococcales
Spirulinales
Gomontiellales
Chroococcidiopsiales
Nostocales

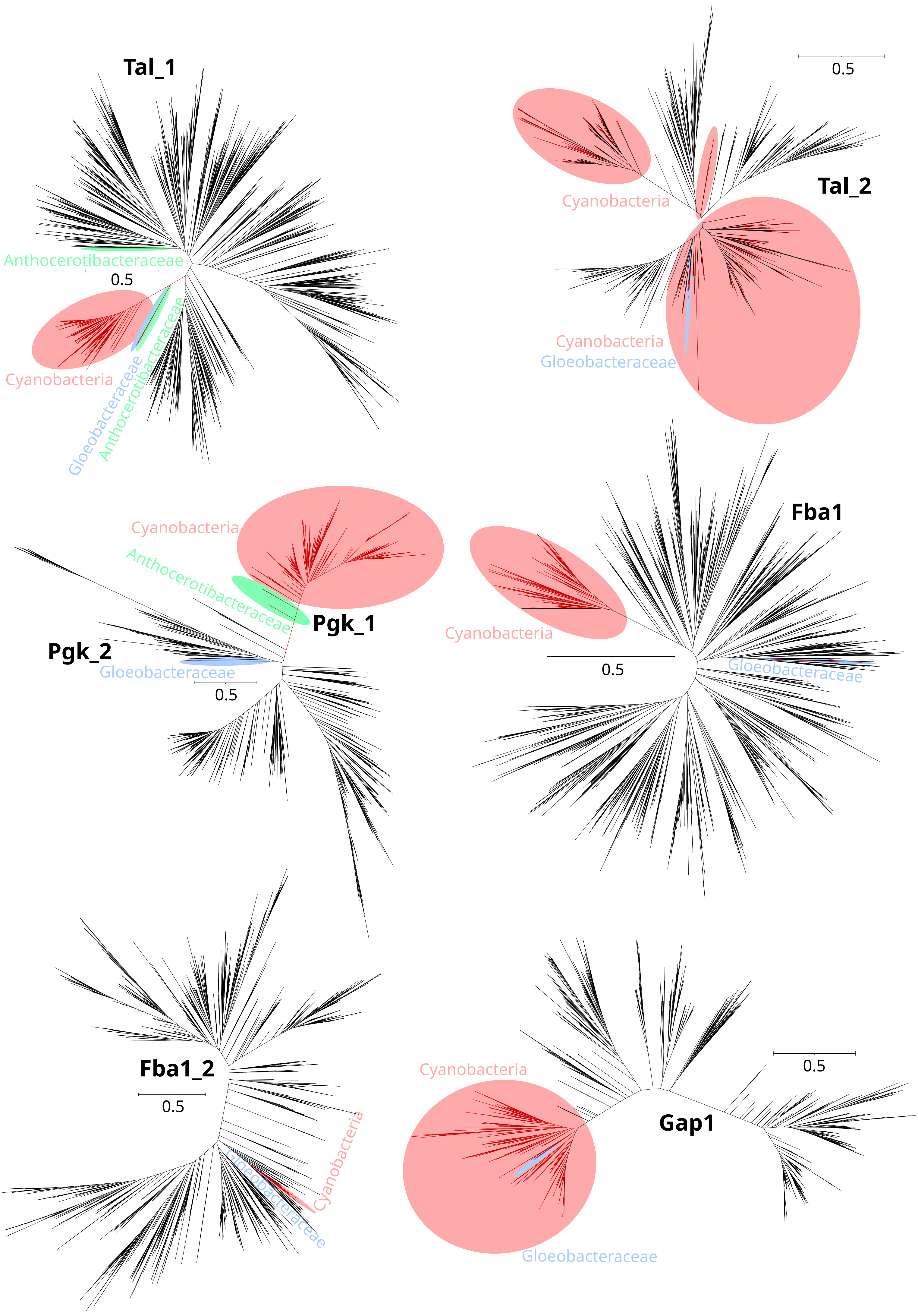
Tal_1
Anthocerotibacteraceae
0.5
Cyanobacteria
Gloeobacteraceae
Anthocerotibacteraceae
0.5
Cyanobacteria
Tal_2
Cyanobacteria
Gloeobacteraceae
Cyanobacteria
Anthocerotibacteraceae
Pgk_1
Pgk_2
Gloeobacteraceae
0.5
Fba1
Cyanobacteria
0.5
Gloeobacteraceae
Fba1_2
0.5
Gloeobacteraceae
Cyanobacteria
Cyanobacteria
0.5
Gap1
Gloeobacteraceae

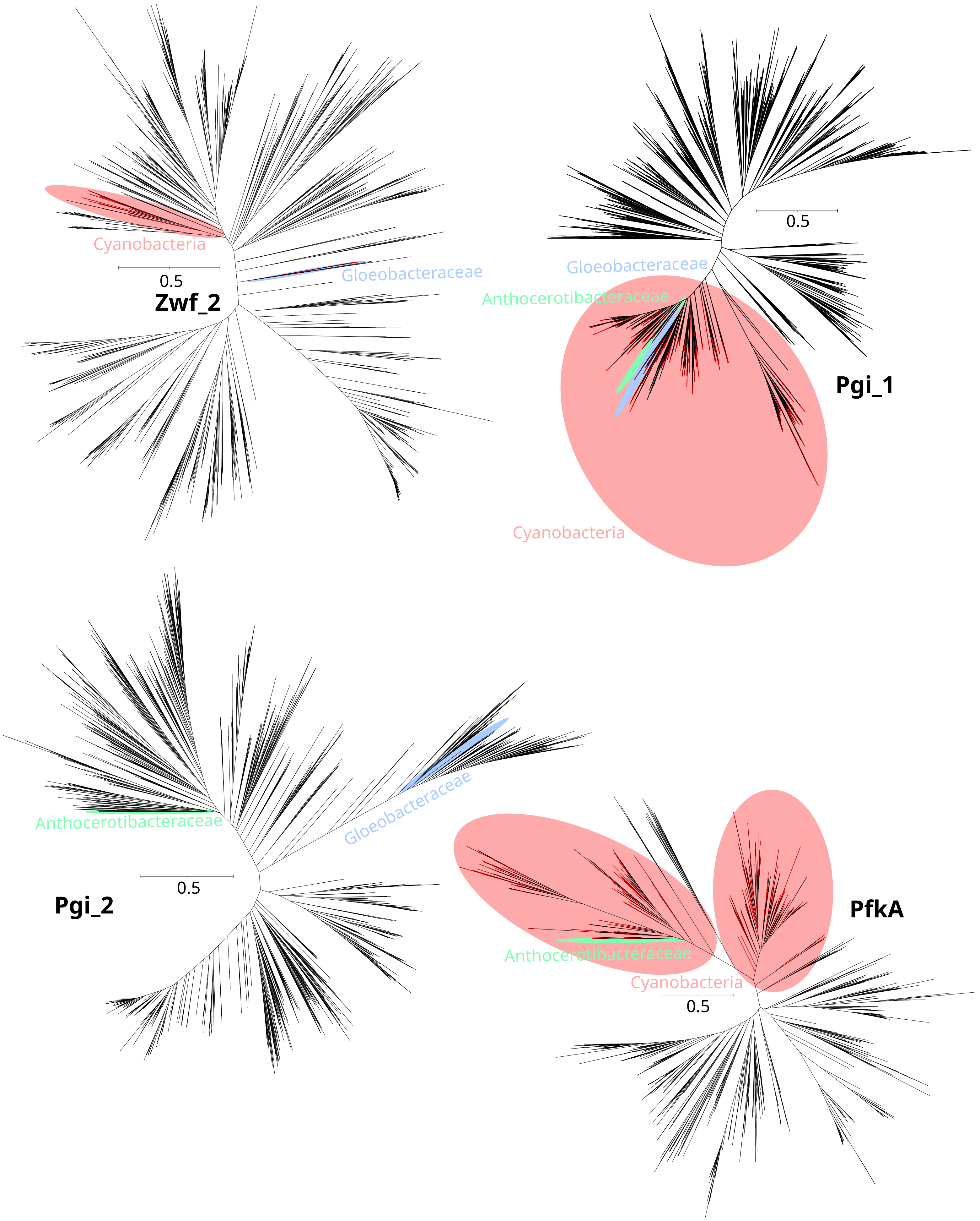
Cyanobacteria
Gloeobacteraceae
0.5
Zwf_2
Gloeobacteraceae
Anthocerotibacteraceae
0.5
Pgi_1
Cyanobacteria
Anthocerotibacteraceae
Gloeobacteraceae
0.5
Pgi_2
Anthocerotibacteraceae
Cyanobacteria
0.5
PfkA

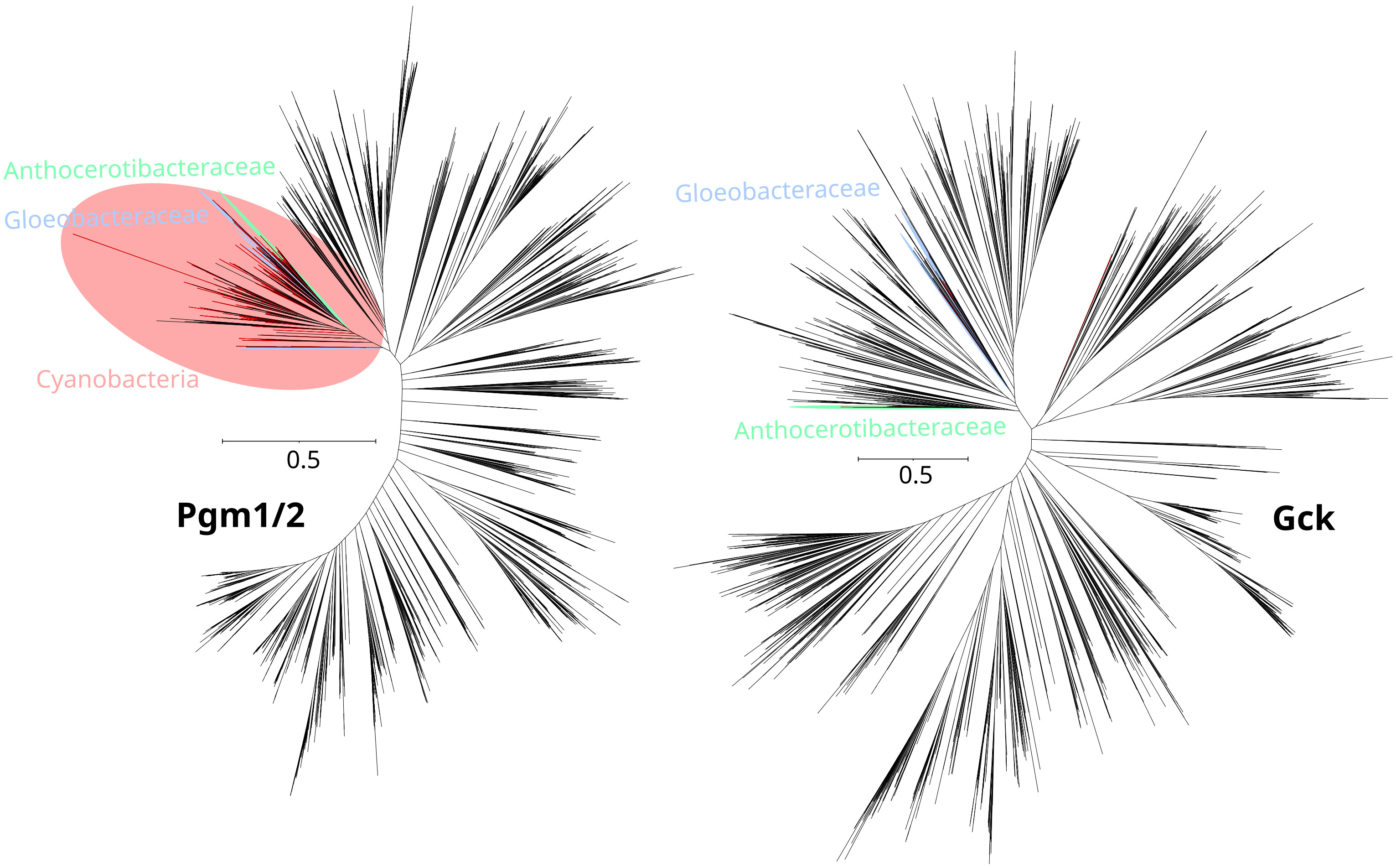
Anthocerotibacteraceae
Gloeobacteraceae
Cyanobacteria
0.5
Pgm1/2
Gloeobacteraceae
Anthocerotibacteraceae
0.5
Gck
